# Atomic layer deposition of ZnO/TiO_2_ nanolaminates as ultra-long life anode material for lithium-ion batteries

**DOI:** 10.1038/s41598-019-48088-2

**Published:** 2019-08-08

**Authors:** Yan-Qiang Cao, Shan-Shan Wang, Chang Liu, Di Wu, Ai-Dong Li

**Affiliations:** National Laboratory of Solid State Microstructures, Jiangsu Key Laboratory of Artificial Functional Materials, Materials Science and Engineering Department, College of Engineering and Applied Sciences, Collaborative Innovation Center of Advanced Microstructures, Nanjing University, Nanjing, 210093 P. R. China

**Keywords:** Batteries, Batteries

## Abstract

In this work, we designed ZnO/TiO_2_ nanolaminates by atomic layer deposition (ALD) as anode material for lithium ion batteries. ZnO/TiO_2_ nanolaminates were fabricated on copper foil by depositing unit of 26 cycles ZnO/26 cycles TiO_2_ repeatedly using ALD. ZnO/TiO_2_ nanolaminates are much more stable than pristine ZnO films during electrochemical cycling process. Therefore, ZnO/TiO_2_ nanolaminates exhibit excellent lithium storage performance with an improved cycling performance and superior rate capability compared to pristine ZnO films. Moreover, coulombic efficiency (CE) of ZnO/TiO_2_ nanolaminates is above 99%, which is much higher than the value of pristine ZnO films. Excellent ultralong-life performance is gained for ZnO/TiO_2_ nanolaminates, retaining a reversible capacity of ~667 mAh g^−1^ within cut-off voltage of 0.05-2.5 V after 1200 cycles of charge-discharge at 500 mA g^−1^. Constructing nanolaminates structures via ALD might open up new opportunities for improving the performance of anode materials with large volume expansion in lithium ion batteries.

## Introduction

Rechargeable lithium ion batteries (LIBs) have attracted great attentions in energy storage area due to their high energy density and benign cycling life^1–4^. However, the current commercially used graphite anode with a relatively low theoretical capacity of 372 mAh g^−1^ cannot satisfy the increasing needs of the ever-enlarging market, especially in hybrid electric vehicles and electric vehicles. Therefore, transition metal oxides with higher theoretical capacities are intensively investigated as alternative anode to graphite^5–10^. Among them, zinc oxide (ZnO) exhibits promising properties, such as environmental benignity, high theoretical capacity (987 mAh g^−1^), as well as higher lithium ion diffusion coefficient compared to other transition metal oxides^11–13^. Nevertheless, ZnO electrodes usually suffer from poor electronic conductivity and huge volume change (228%) during lithiation and delithiation, resulting in poor electrochemical reaction kinetics and severe pulverization along with inferior cyclic stability and rate capability^14,15^. Therefore, rational design of high performance ZnO based anode for LIBs still remains a challenge. To this regard, great efforts have been devoted to improving the performance of ZnO anode, such as construction of various nanostructured electrodes and modification of ZnO^16–21^. For example, Xie *et al*. reported a ZnO based nanostructured anode of sandwich-like Ag-C@ZnO-C@Ag-C hybrid hollow microspheres, which exhibits a large reversible capacity of 1670 mAh g^−1^ after 200 cycles at a current density of 200 mA g^−1^ with excellent high-rate performance. The special structural features, including hollow structures, the sandwich-like shells, and the nanoscale dimension, contribute to the outstanding electrochemical performance^22^.

Atomic layer deposition (ALD) is a novel and promising thin film deposition technique based on sequential self-limited and complementary surface chemisorption reactions, which is able to deposit ultrathin, uniform, and conformal layers with precise thickness control^23,24^. This novel method has shown great prospects in preparation and modification of materials in energy area^25,26^, including anodes^27,28^, cathodes^29,30^, solid electrolytes^31–33^ of Li-ion batteries, as well as supercapacitors^34^. ALD has been used to improve the performance of ZnO anodes via depositing active ZnO onto carbon based supporters (graphene, carbon black, carbon foam, etc.)^12,35^ and surface modification^36^. For example, Zhao *et al*. reported that 3D carbon/ZnO nanomembrane foam prepared by ALD can retain 92% capacity after 700 cycles at 2 A g^−1^ and deliver a remarkable areal capacity of 4.3 mAh cm^−2^^35^. Lu *et al*. fabricated ZnO-carbon black nanocomposites by directly depositing ZnO on carbon black using ALD, which exhibit excellent cyclic stability with a specific capacity of 1026 mAh g^−1^ maintained after 500 cycles^12^. Shi *et al*. utilized ALD Al_2_O_3_ coating to stabilize ZnO-graphene anode, which can maintained a reversible specific capacity of ~490 mAh g^−1^ after 100 cycles^36^.

Besides, ALD is a very powerful technique to construct novel nanostructures, various nanostructured electrodes have been designed and fabricated by ALD^37^. Nanolaminates are composite films consisted of alternating layers of different materials with individual layer thicknesses down to nanometer scale. ALD is ideally suited for fabricating nanolaminate films due to its precise thickness control of ~1 Å for the individual layers in the composites^38^. Herein, we designed the novel ZnO/TiO_2_ nanolaminates as anode materials for LIBs. ZnO/TiO_2_ nanolaminates were fabricated directly on copper foil by depositing 24 units of 26 cycles ZnO/26 cycles TiO_2_ repeatedly using ALD. Herein, ZnO is the main active material, providing high capacity. While, the inserted TiO_2_ layers are designed to stabilize ZnO from the following two considerations. Firstly, TiO_2_ can divide thick ZnO into multi-nanolayers, the dimension (thickness) of each ZnO layer is reduced to nanometer scale. Secondly, TiO_2_ is stable during charging-discharging process with the volume change of only 4%^39,40^. Therefore, the stable TiO_2_ thin films can also act as protective layers for ZnO. Accordingly, the electrochemical tests demonstrate that the reversible capacity and the rate performance of ZnO films are greatly improved after inserting TiO_2_ thin films. Moreover, ZnO/TiO_2_ nanolaminates exhibit excellent ultralong-life performance, retaining a reversible capacity of ~667 mAh g^−1^ almost without decay after 1200 cycles of charge-discharge at 500 mA g^−1^ with upper cut-off voltage of 2.5 V. Therefore, ZnO/TiO_2_ nanolaminates can work as ultra-long lifespan anodes in LIBs.

## Results

**Figure 1:**
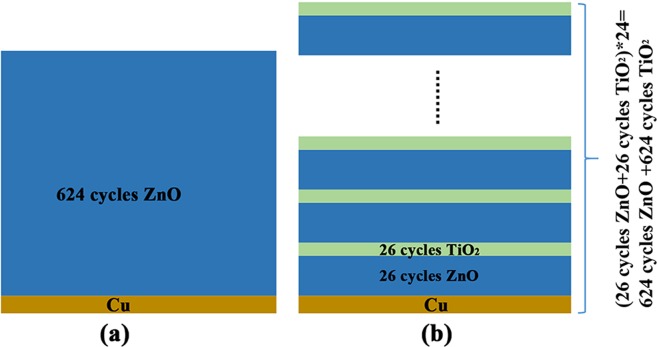
Schematic structure of (**a**) pristine ZnO films and (**b**) ZnO/TiO_2_ nanolaminates prepared by ALD.

ZnO/TiO_2_ nanolaminates with ZnO/TiO_2_ thickness ratio of ~5:1 was designed. Considering the growth rate per cycle (GPC) is around 1.75 Å for ZnO and 0.36 Å for TiO_2_ in our system^41,42^, the nanolaminate was fabricated by depositing 24 alternate layers of ZnO (26 cycles) and TiO_2_ (26 cycles) with ZnO as the beginning layer. The schematic of ALD deposition process for ZnO/TiO_2_ nanolaminates is shown in Fig. S1. For comparison, the control sample of pristine ZnO film (624 cycles ZnO) was also prepared. Both samples were deposited directly onto Cu foil. The structure of samples are showed in Fig. 1.

**Figure 2:**
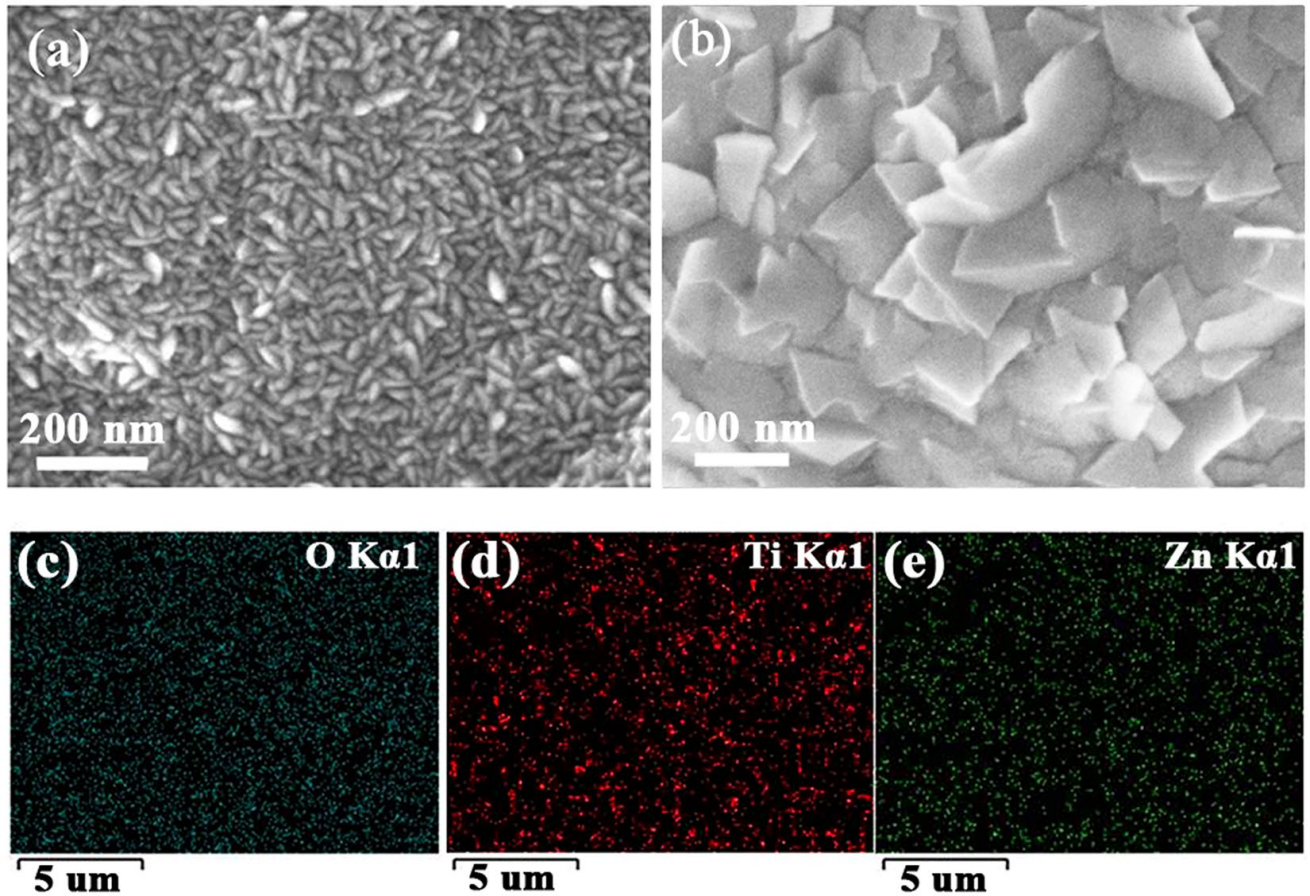
SEM images of (**a**) pristine ZnO films and (**b**) ZnO/TiO_2_ nanolaminates, (**c**–**e**) SEM element mapping results of ZnO/TiO_2_ nanolaminates.

SEM was performed to observe the surface morphology of pristine ZnO films and ZnO/TiO_2_ nanolaminates. In Fig. 2(a), it can be seen that pristine ZnO films deposited by ALD exhibit a lot of spindly grains with ~15 nm × 55 nm. After inserting TiO_2_ interlayer, ZnO/TiO_2_ nanolaminates show distinct morphology with much larger grain size of ~170 nm, as shown in Fig. 2(b). The grain size distribution is presented in Fig. S2 and S3. Due to the excellent conformality and uniformity of ALD, both films can cover the Cu foil conformally. EDS measurement in Fig. S4 provides the evidence for the presence of Zn, Ti, and O elements. In addition, the element mappings by EDS for the ZnO/TiO_2_ nanolaminates are also conducted, as shown in Fig. 2(c–e), which indicate that Zn, Ti and O elements are uniformly distributed over the whole surface. Moreover, the thickness is determined to be around 104 nm and 132 nm for pristine ZnO films and ZnO/TiO_2_ nanolaminates, respectively, from the cross-section FESEM images (Fig. S5). The crystallinity of as deposited ZnO and ZnO/TiO_2_ nanolaminates were also characterized by XRD, as shown in Fig. S6. It can be seen that as-deposited ZnO shows a weak (002) peak from hexagonal wurtzite ZnO. However, no peaks assigned to ZnO or TiO_2_ can be observed for ZnO/TiO_2_ nanolaminates. Inserting amorphous Al_2_O_3_ layer can inhibit the ZnO crystal growth has been reported by Elam *et al*.^38^. Therefore, amorphous TiO_2_ here can also reduce the crystallinity of ZnO.

**Figure 3:**
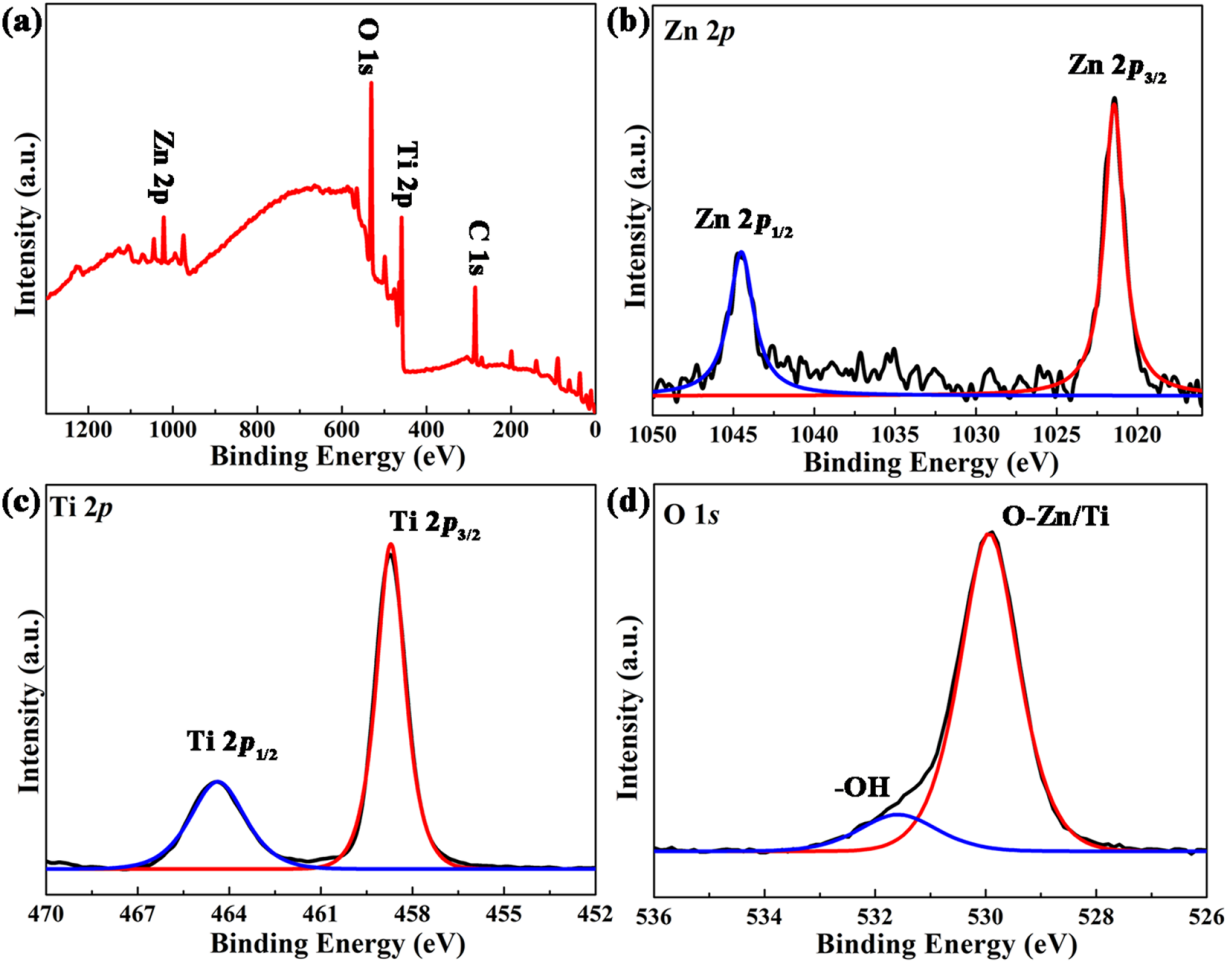
(**a**) The XPS survey spectra, high resolution (**b**) Zn 2p, (**c**) Ti 2p, and (**d**) O 1 s XPS spectra of ZnO/TiO_2_ nanolaminates.

The composition and chemical state of the ZnO/TiO_2_ nanolaminates were evaluated via XPS. The Zn 2p, Ti 2p and O 1 s signals centred at 1021.5, 458.7, and 529.9 eV can be easily found in the survey spectrum, revealing the coexistence of Zn, Ti and O in ZnO/TiO_2_ nanolaminates (Fig. 3a). In Fig. 3(b), Zn 2p exhibits two distinct peaks at 1044.5 eV and 1021.5 eV, which can be assigned to Zn 2p_1/2_ and Zn 2p_3/2_ peaks of Zn-O bonding with the spin orbit splitting energy of 23.0 eV, in good agreement with the value for ZnO^43^. In Fig. 3(c), the doublet with at 464.4 eV and 458.7 eV corresponds to Ti 2p_1/2_ and Ti 2p_3/2_ of Ti-O bonding with the spin orbit splitting energy of 5.7 eV, consistent with the value of TiO_2_^44^. The spectrum of O 1 s (Fig. 3d) shows the main peak for O-Zn/O-Ti bonds of ZnO/TiO_2_ at 529.9 eV, the position of O-Zn and O-Ti are too close to be distinguished^45,46^. Besides, the peak related to -OH on the surface of ZnO/TiO_2_ nanolaminates at 531.6 eV can also be detected^47^. Both SEM EDS and XPS spectra can confirm that ZnO and TiO_2_ coexist in the films. In addition, the Zn/Ti atom distribution was also measured by XPS depth profile, as shown in Fig. S7, the multilayer structure of ZnO/TiO_2_ nanolaminates can be recognized. Cross-sectional TEM images also confirm the layer-by-layer structure of ZnO/TiO_2_ nanolaminates, as shown in Fig. S8.

**Figure 4:**
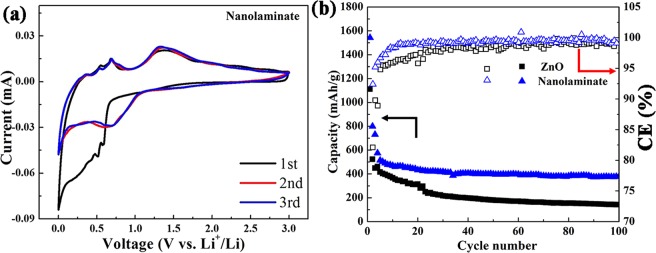
CV curves of (**a**) ZnO/TiO_2_ nanolaminates electrodes for the initial three cycles at the scan rate of 0.3 mV/s in the voltage range of 0–3 V. (**b**) Cycling performance and CE of ZnO/TiO_2_ nanolaminates and pristine ZnO films at 500 mA g^−1^ for 100 cycles in the potential range of 0.05–2.0 V.

Figure 4(a) shows the cyclic voltammograms (CV) of ZnO/TiO_2_ nanolaminates anode for the initial three cycles. Based on the previous literatures, the electrochemical process of ZnO towards lithium can be described as the following reactions^48^:

1 $${\rm{ZnO}}+2{{\rm{Li}}}^{+}+2{{\rm{e}}}^{-}\leftrightarrow {{\rm{Li}}}_{2}{\rm{O}}+{\rm{Zn}}$$

2 $${\rm{Zn}}+{{\rm{xLi}}}^{+}+{{\rm{xe}}}^{-}\leftrightarrow {{\rm{Li}}}_{{\rm{x}}}{\rm{Zn}}\,({\rm{x}}\le 1)$$

For ZnO/TiO_2_ nanolaminates, there are two small shoulders at ~0.59 V and 0.51 V as well as a broad peak centred at ~0.30 V can be discerned during the first cathodic scan. The shoulder at 0.59 V is derived from the decomposition of liquid electrolyte to form a solid electrolyte interphase (SEI) layer^49,50^. According to previous reports, the conversion reaction between ZnO and Li ion to generate Zn and Li_2_O generally takes place at ~0.50 V, while the subsequent alloying reaction between Zn and Li ion to produce Li_x_Zn alloys at around 0.25 V^11^. Two above-mentioned electrochemical reactions overlap partially here, therefore, a broad reduction peak centred at ~0.30 V and a small shoulder near 0.51 V can be observed. This phenomenon can be commonly observed in many other ZnO-based anodes^22,36,51^. In the subsequent first anodic scan, three peaks located between 0.20–0.80 V (0.36 V, 0.53 V and 0.79 V) are ascribed to the multistep dealloying process of Li_x_Zn alloy^13^. Another broad peak at 1.32 V can be related to the formation of ZnO by the redox reaction between Li_2_O and Zn^52^. In the subsequent two cycles, the CV curves show very good reproducibility, indicating high reversibility and excellent cycling stability of ZnO/TiO_2_ nanolaminates. The CV curves of pristine ZnO films shown in Fig. S9 are similar to those of ZnO/TiO_2_ nanolaminates. However, pristine ZnO films exhibit worse cycling stability than ZnO/TiO_2_ nanolaminates, as indicated with reduced enclosed CV area. In addition, pristine ZnO films exhibit an irreversible anodic peak at ~2.7 V, corresponding to the oxidation process of Zn back to ZnO. This peak disappears after 2 cycles, indicating the conversion reaction is irreversible for ZnO, same phenomenon has been observed in previous literature^53^.

Figure 4(b) plots the cycling performance of ZnO/TiO_2_ nanolaminates and pristine ZnO films at 500 mA g^−1^ for 100 cycles in the potential range of 0.05–2.0 V. It can be seen that the capacity of ZnO/TiO_2_ nanolaminates only drops in the initial few cycles, and then maintains a relatively stable capacity. Therefore, ZnO/TiO_2_ nanolaminates can maintain a reversible capacity of 381 mAh g^−1^ after 100 cycles at 500 mA g^−1^, demonstrating its excellent cycling performance. In contrast, pristine ZnO films exhibit a decreasing capacity along with cycling, only about 141 mAh g^−1^ can be remained after 100 cycles. Obviously, the cycling performance of ZnO/TiO_2_ nanolaminates is much better than pristine ZnO films. Fig. S10 exhibits the discharge/charge profiles ZnO and ZnO/TiO_2_ nanolaminates at 500 mA g^−1^ with cut-off potential of 0.05–2.0 V in 1^st^, 2^rd^, 3^th^, 50^th^ and 100^th^ cycle. More importantly, the CE of ZnO/TiO_2_ nanolaminates is above 99%, which is much higher than the value of pristine ZnO films. Especially in first 20 cycles, for example, the CE at 15^th^ cycle is 99.0% and 96.2% for ZnO/TiO_2_ nanolaminates and pristine ZnO films, respectively. The cycling performance was also conducted at the current density of 200 mA g^−1^, as shown in Fig. S11 and S12, which shows the similar phenomena.

**Figure 5:**
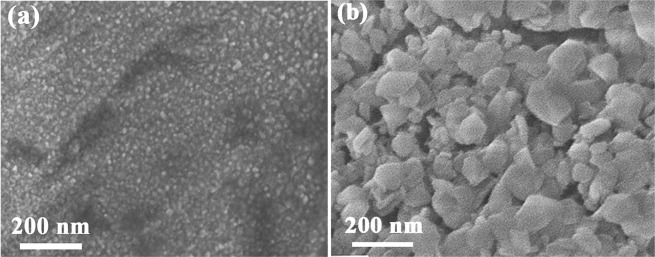
SEM images of (**a**) pristine ZnO films and (**b**) ZnO/TiO_2_ nanolaminates after 100 cycles charging-discharging at 200 mA g^−1^.

In order to explore the mechanism of enhanced electrochemical performance, SEM was performed to observe the morphology change after charging-discharging process. Figure 5(a) shows the SEM image of pristine ZnO films after 100 cycles cycling at the current density of 200 mA g^−1^. It can be seen that the spindly grains of ZnO are disappeared after cycling, leaving lots of small particles with the size less than 10 nm. It implies that the pulverization is very severe for pristine ZnO film during cycling. It has been demonstrated in previous reports that ZnO materials possess large volume expansion (228%) upon cycling, which would lead to severe pulverization for ZnO^11,13^. In contrast, the morphology of ZnO/TiO_2_ nanolaminates is much more stable than pristine ZnO, as shown in Fig. 5(b). Although the pulverization of grains still can be observed after cycling, large grain of ZnO/TiO_2_ nanolaminates is almost retained, suggesting its superior structural stability. Even cycled at high current density of 500 mA g^−1^ for 100 cycles, ZnO/TiO_2_ nanolaminates can still maintain its structure, as shown in Fig. S13. In addition, EDS was also conducted for ZnO/TiO_2_ nanolaminates after 100 cycling of 500 mA g^−1^, as shown in Fig. S14. It can be seen that Zn, Ti, O elements are still uniformly distributed after cycling. It can be demonstrated that ZnO/TiO_2_ nanolaminates are much more stable than pristine ZnO films.

**Figure 6:**
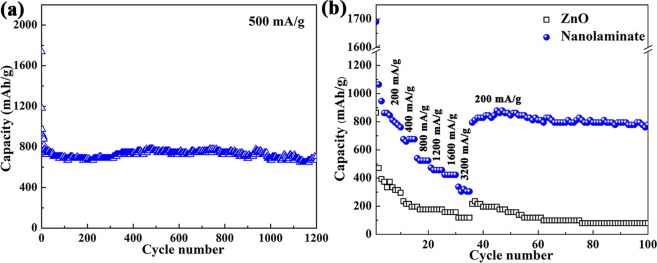
(**a**) Ultra-long cycling performance of ZnO/TiO_2_ nanolaminates at 500 mA g^−1^ for 1200 cycles in the potential range of 0.05–2.5 V. (**b**) Comparison of rate capability for ZnO/TiO_2_ nanolaminates and pristine ZnO films anodes in the potential range of 0.05–2.5 V.

Furthermore, we also explore the effect of cut-off voltage on the performance of ZnO/TiO_2_ nanolaminates. Therefore, cycling performance was also conducted in the potential range of 0.05–2.5 V for comparison, as shown in Fig. S15. It can be easily seen that larger cycling potential range delivers higher capacity. Moreover, ZnO/TiO_2_ nanolaminates can still show great cycling stability, exhibiting a reversible capacity of 875 and 706 mAh g^−1^ after 100 cycles at 200 and 500 mA g^−1^, respectively. Therefore, long-life cycling and rate capability were also performed in the potential range of 0.05–2.5 V. Figure 6(a) plots the long-life cycling performance conducted at 500 mA g^−1^ for 1200 cycles within the voltage of 0.05–2.5 V. The capacity only decreases at the initial several cycles. After that, the reversible capacity can be stabilized at ~667 mAh g^−1^ almost without decay, revealing a remarkable long-life performance. The rate capability of ZnO/TiO_2_ nanolaminates was also evaluated, as shown in Fig. 6(b), which was performed from 200 mA g^−1^ to 3200 mA g^−1^. It can be clearly seen that the ZnO/TiO_2_ nanolaminates exhibit excellent capacity retention at various current densities. Even at a high current density of 3200 mA g^−1^, a high capacity of ~307 mAh g^−1^ can be achieved, demonstrating a superior high-rate performance of ZnO/TiO_2_ nanolaminates. Furthermore, the cell is able to deliver a reversible capacity of 750 mAh g^−1^ when the current density returns back to 200 mA g^−1^. While pristine ZnO films show very poor rate ability.

**Figure 7:**
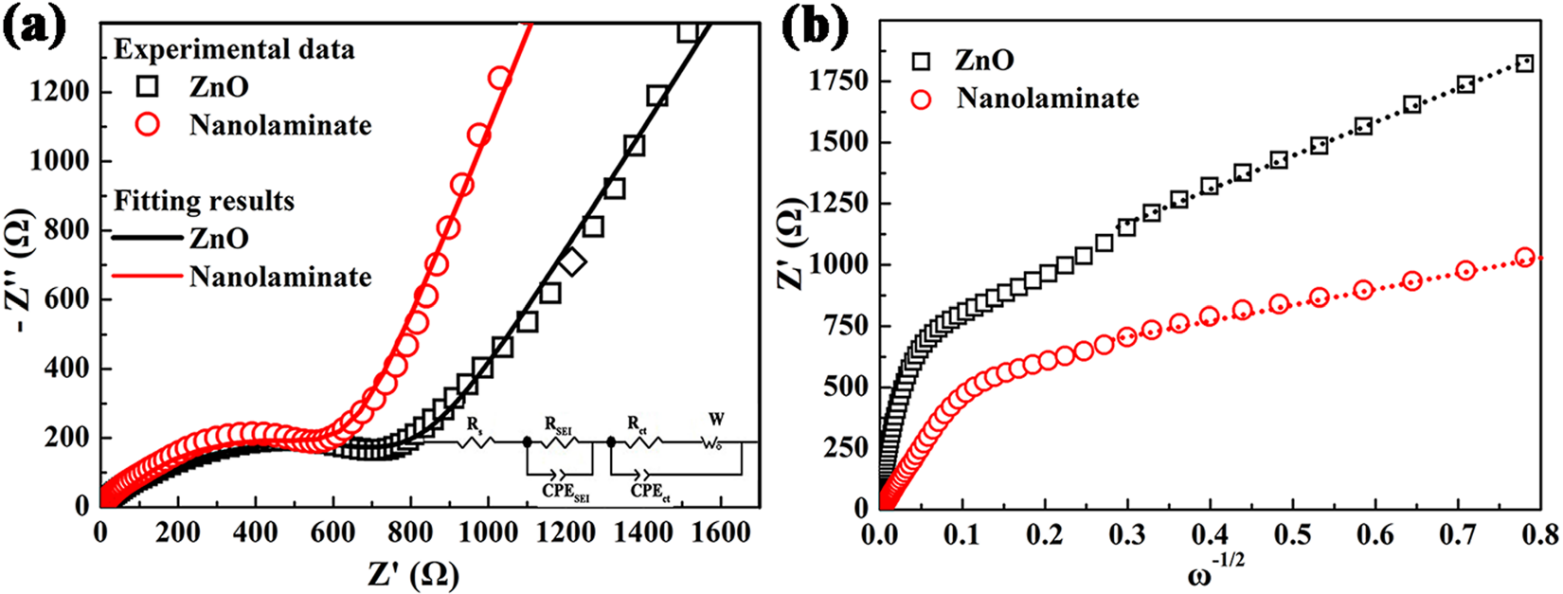
(**a**) Nyquist plots and (**b**) Randles plots of ZnO films and ZnO/TiO_2_ nanolaminates. The insert of (a) is the equivalent circuit model for EIS fitting.

To explore the charge transfer and Li ion diffusion characteristics in the pristine ZnO films and ZnO/TiO_2_ nanolaminates, EIS measurements were further conducted. Figure 7(a) compares the Nyquist plots of ZnO films and ZnO/TiO_2_ nanolaminates. Obviously, both Nyquist plots exhibit a semicircle in the high-middle frequency region and a slop line in the low frequency region. According to the reported literatures, the semicircle in high-frequency region is ascribed to the formation of the SEI layer and contacting impedance between active materials and electrolyte, the semicircle in the middle frequency range is assigned to the charge-transfer resistance (R_ct_) of electrode/electrolyte interface. The slopping line in low frequency region corresponds to diffusion of Li ion in electrodes^54^. Therefore, the EIS data were fitted using the mode of the insert in Fig. 7(a)^55^. Herein, R_s_, R_SEI_, and R_ct_ represent electrolyte resistance, SEI resistance, and charge-transfer resistance, respectively. CPE is the respective constant-phase element contributing to the semicircle in the experimental spectra. And W is the Warburg impedance. The fitted data are listed in Table 1. The SEI resistance and the charge-transfer resistance of the ZnO/TiO_2_ nanolaminates are lower than pristine ZnO films. Furthermore, Randles plots (Warburg impedance (Z_w_) vs. ω^−1/2^) of both electrodes were built, as shown in Fig. 7(b), where the slope of the fitted line in low frequency region is the Warburg coefficient (σ_w_)^56^. The diffusion coefficient of Li ion (D_Li_ + ) is inversely proportional to σ_w_^2^^57,58^. Obviously, the value of σ_w_ for ZnO/TiO_2_ nanolaminates electrodes is smaller than that of ZnO films electrodes, thereby possessing higher D_Li_+. It is observed that the Warburg coefficients (σ_w_) of ZnO films and ZnO/TiO_2_ nanolaminates electrodes are 1394.0 and 642.6, respectively. The EIS measurements indicate that the formation of ZnO/TiO_2_ nanolaminates can decrease the SEI resistance and charge-transfer resistance, as well as improve the solid state Li ion diffusion in ZnO electrode. Accordingly, the superior lithium storage characteristics are exhibited in ZnO/TiO_2_ nanolaminates electrode compared to pristine ZnO films.

**Table 1:** EIS fitting data of pristine ZnO films and ZnO/TiO_2_ nanolaminate electrodes.

Electrode	R_s_ (Ω)	R_SEI_ (Ω)	R_ct_ (Ω)	σ_w_
ZnO	3.5	25.1	804.5	1394.0
Nanolaminate	2.8	19.6	590.2	642.6

The excellent electrochemical performance of ZnO/TiO_2_ nanolaminates as anodes of LIBs can be ascribed to the following factors. (1) The dimension (thickness) of each active ZnO layer is reduced to nanometer scale. (2) Moreover, each active ZnO layers are protected by stable thin TiO_2_ layers from both side. Both above two factors can contribute to improving the stability of ZnO, therefore a remarkable electrochemical performance can be achieved.

## Conclusions

In summary, a novel nanostructured anode of ZnO/TiO_2_ nanolaminates was developed in this work, which was constructed by depositing unit of 26 cycles ZnO/26 cycles TiO_2_ repeatedly using ALD. It is demonstrated that ZnO/TiO_2_ nanolaminates are much stable than pristine ZnO films during electrochemical cycling process. Accordingly, the electrochemical tests demonstrate that the reversible capacity and the rate performance of ZnO films are greatly improved after inserting TiO_2_ thin films. Moreover, ZnO/TiO_2_ nanolaminates exhibit excellent ultralong-life performance, retaining a reversible capacity of ~667 mAh g^−1^ in potential range of 0.05–2.5 V almost without decay after 1200 cycles of charge-discharge at 500 mA g^−1^. In addition, EIS measurements indicate that the formation of ZnO/TiO_2_ nanolaminates can decrease the SEI resistance and charge-transfer resistance, as well as improve the solid state Li ion diffusion in bulk electrode. Above results imply that constructing nanolaminates structures via ALD might open up new opportunities for improving the performance of anode materials with large volume change in LIBs. ALD exhibits excellent large area uniformity, conformality, and precise thickness control. Therefore, it is very suitable for large scale fabrication. However, the cost of ALD may be too expensive to be used in LiBs for active materials deposition at present. Nowadays, various new type of ALD are researched to solve the cost problem, such as spatial ALD. We believe that ALD will play an important role in energy area in soon future.

## Methods

### Fabrication of ZnO/TiO_2_ nanolaminates

In ALD process, diethyl zinc (DEZ, 6N, Nata Opto-electronic Material Co., Ltd), titanium tetrachloride (TiCl_4_, 5N, Suzhou Fornano Corporation Ltd.) and deionized water were used as Zn, Ti precursors and oxygen source, respectively. All the precursors were kept at room temperature. Pure N_2_ (5N) was used as carrier and purge gas. ALD process was performed at 130 °C in a commercial Picosun SUNALE^TM^ R-200 ALD reactor. ZnO/TiO_2_ nanolaminates were fabricated through an alternate deposition of ZnO and TiO_2_ by ALD. Pulse time of three precursors was 0.1 s with a 4 s N_2_ purging step to remove the redundant reactants and by-products. In our ALD systems, the growth rate per cycle (GPC) is around 1.75 Å for ZnO and 0.36 Å for TiO_2_^41,42^. Herein, we designed the ZnO/TiO_2_ nanolaminates with ZnO/TiO_2_ thickness ratio of around 5:1. Therefore, the nanolaminate was fabricated by depositing 24 alternate layers of ZnO (26 cycles) and TiO_2_ (26 cycles) with ZnO as the beginning layer. For comparison, the control sample of pristine ZnO film (624 cycles ZnO) was also prepared. Both samples were deposited directly onto Cu foil.

### Materials characterizations

The surface chemical features were investigated by X-ray photoelectron spectroscopy (XPS, Thermo Fisher K-Alpha) with standard Al Kα (1486.7 eV) X-ray source. The binding energies were calibrated using the signal from the adventitious carbon (binding energy C 1 s = 284.6 eV).

XPS spectra were fitted using Gaussian–Lorentzian functions. XPS depth profile was obtained by performing the XPS elemental scan after each 30 s Ar ion of 1000 eV etching. The resolution and sensitivity of XPS instrument is 0.35 eV and 3.7 kcps, which is measured by the full width at half maximum (FWHW). The microstructure and morphology were examined by field emission scanning electron microscopy (FESEM, Ultra 55, ZEISS) in InLens mode with voltage of 3 kV. Energy-dispersive X-ray spectroscopy (EDS) were performed to explore the element distribution in the same SEM system with the voltage of 18 kV. Crystallinity of thin films on Cu foil was analysed by a Rigaku-D/MAX 2000x-ray diffraction (XRD) system with Cu Kα radiation. Transmission electron microscopy (TEM, Tecnai F20 S-Twin, FEI) was conducted to observe the layer-by-layer structure of nanolamintes. The sample for TEM cross-section characterization was prepared by the combination of mechanical grinding and ion beam thinning.

### Electrochemical measurements

2032-type coin half-cells were used for the electrochemical measurements. The pristine ZnO films or ZnO/TiO_2_ nanolaminates on copper foil were directly used as the working electrodes. A metallic lithium foil served as both the counter electrode and the reference electrode. The porous polypropylene film (Celgard 2500) was used as the separator. A solution of 1 M LiPF_6_ dissolved in a mixture of ethylene carbonate (EC) and dimethyl carbonate (DEC) (1:1, w/w) was used as liquid electrolyte. The coin cells were assembled in an argon-filled glove box, in which oxygen and water contents were less than 1 ppm. The galvanostatic charge-discharge tests were performed using a battery testing system (LAND CT2001A) at various current densities in the potential range between 0.05 V–2.0 V and 0.05 V−2.5 V. Cyclic voltammetry evaluations (CV) were conducted at the scanning rate of 0.3 mV/s at a voltage between 0 V–3 V using an electrochemical workstation (CHI 660E). Electrochemical impedance spectroscopy (EIS) were taken on the same electrochemical workstation by applying an AC voltage amplitude of 5 mV in the frequency range of 10 mHz ~100 kHz on the open circuit voltage of the cells.

## Supplementary information

Supplementary information
