# Supplementary material for: Atomic layer deposition of ZnO/TiO2 nanolaminates as ultra-long life anode material for lithium-ion batteries
Source: Sci Rep. 2019 Aug 8;9:11526. doi: 10.1038/s41598-019-48088-2 (PMC6687889; doi:10.1038/s41598-019-48088-2)
Supplement: Supplementary file 1 — Supplementary information [file 41598_2019_48088_MOESM1_ESM.pdf]

Supplementary materials

Atomic layer deposition of ZnO/TiO_2 nanolaminates as ultra-long life anode material for lithium-ion batteries

Yan-Qiang Cao, Shan-Shan Wang, Chang Liu, Di Wu and Ai-Dong Li*

National Laboratory of Solid State Microstructures, Jiangsu Key Laboratory of Artificial Functional Materials, Materials Science and Engineering Department, College of Engineering and Applied Sciences, Collaborative Innovation Center of Advanced Microstructures, Nanjing University, Nanjing 210093, P. R. China.

Correspondence and requests for materials should be addressed to A.D. L. (adli@nju.edu.cn)

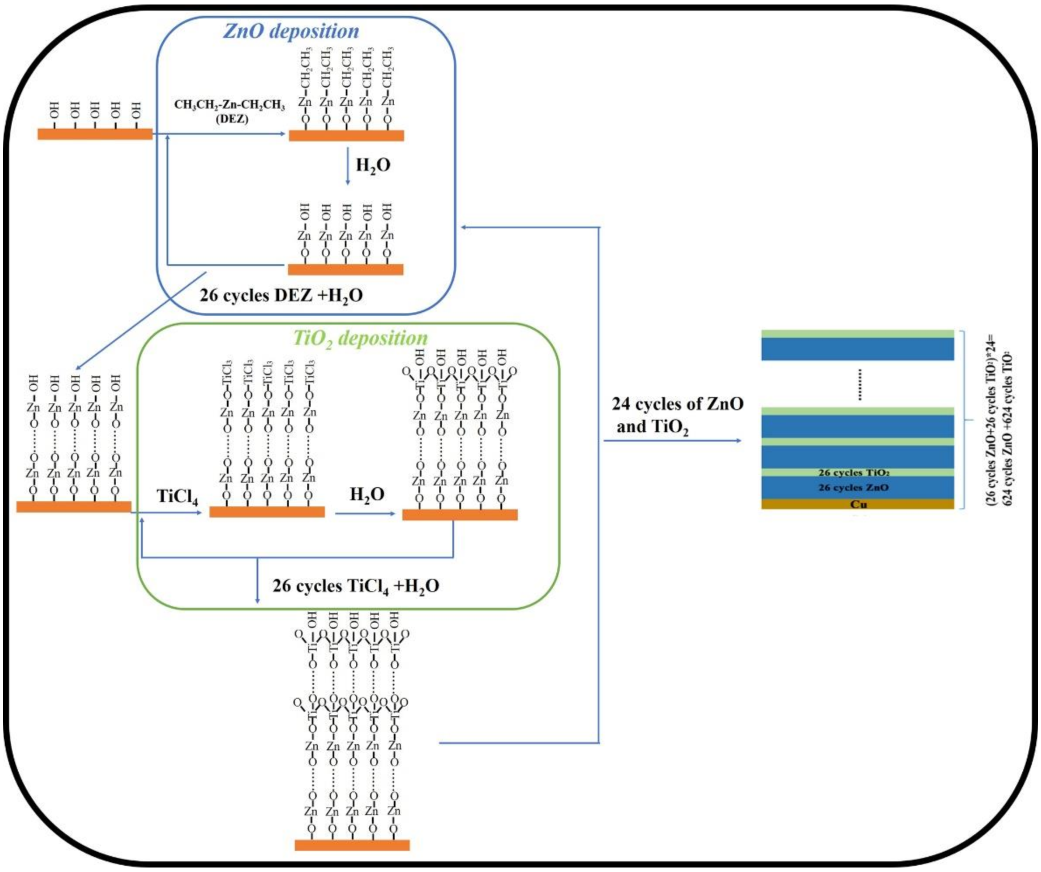

Figure S1. The schematic of ALD deposition process for ZnO/TiO_2 nanolaminates.

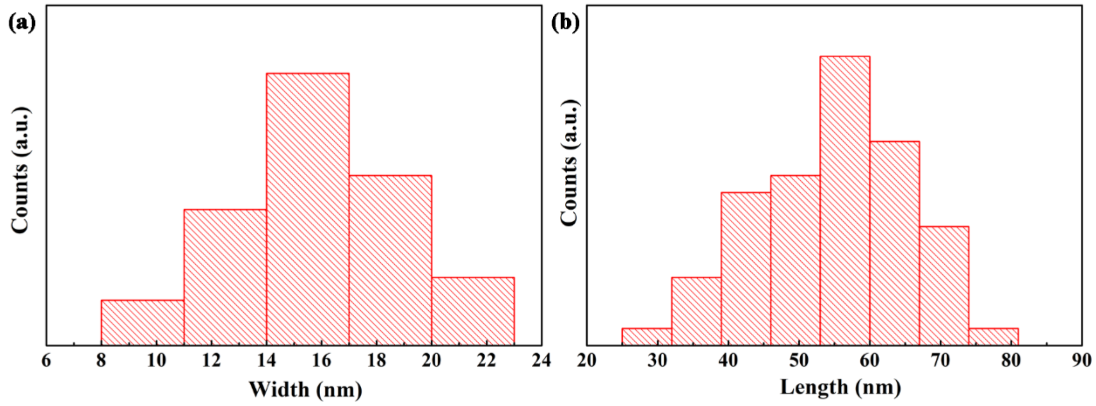

Figure S2. (a) Width and (b) length distribution of spindly grains for pure ZnO.

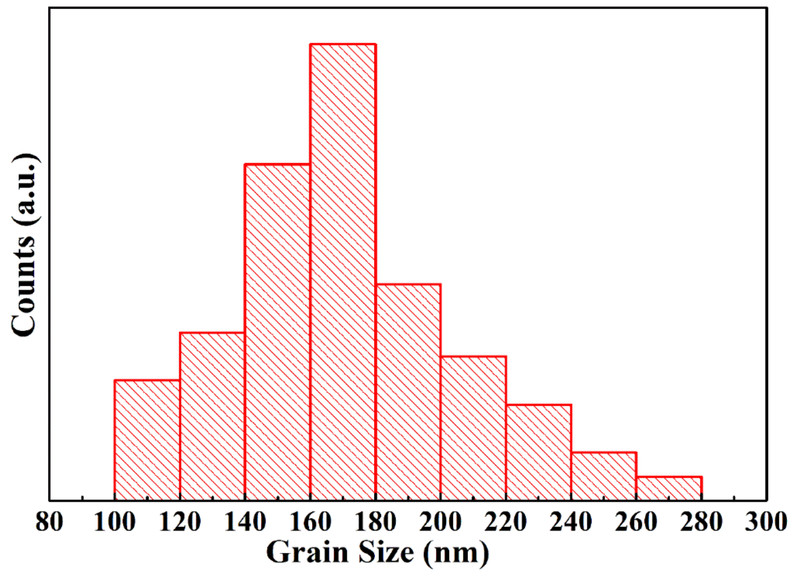

Figure S3. Grain size distribution of ZnO/TiO_2 nanolaminates.

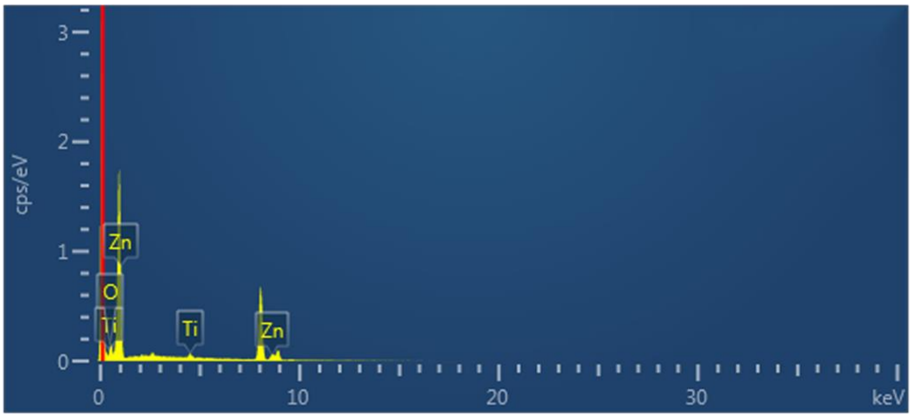

Figure S4. EDS spectrum of ZnO/TiO_2 nanolaminates.

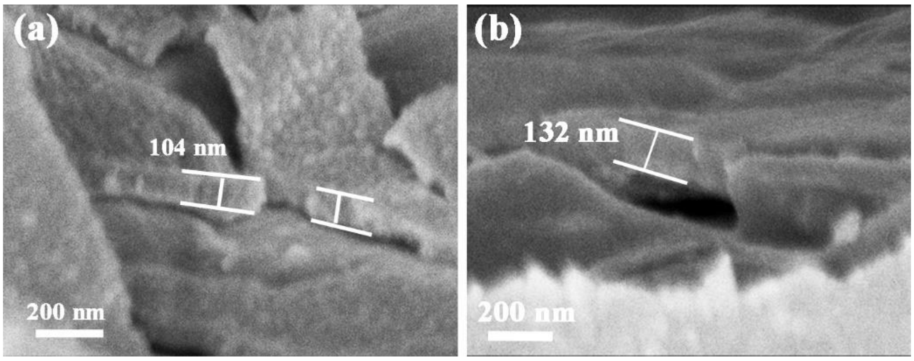

Figure S5. Cross-section SEM images of (a) pristine ZnO films and (b) ZnO/TiO_2 nanolaminates.

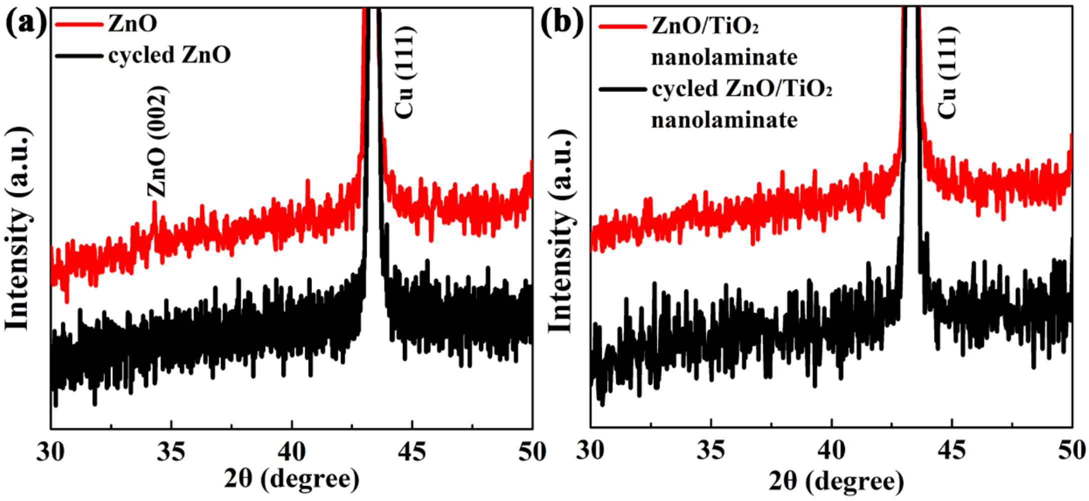

Figure S6. XRD spectra of (a) pure ZnO and (b) ZnO/TiO_2 nanolaminates before and after cycling

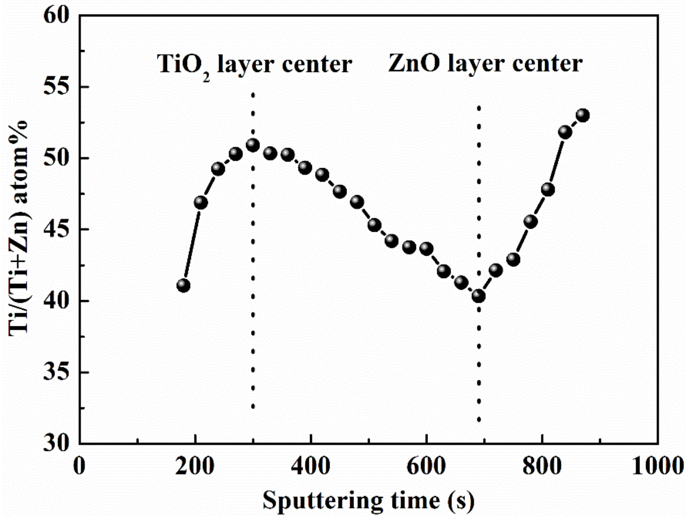

Figure S7. XPS depth profile of Ti/(Ti+Zn) atom% for ZnO/TiO_2 nanolaminates.

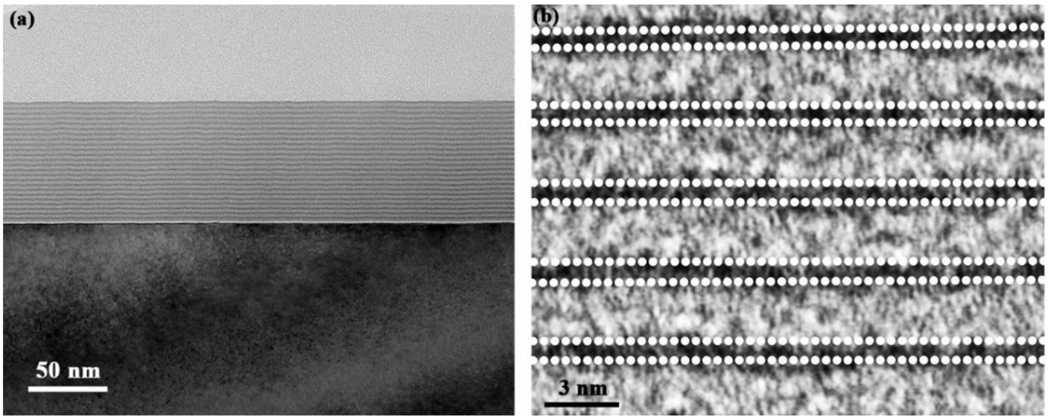

Figure S8. TEM images of (16 cycles ZnO/16 cycles TiO_2)*24 nanolaminates with different magnification.

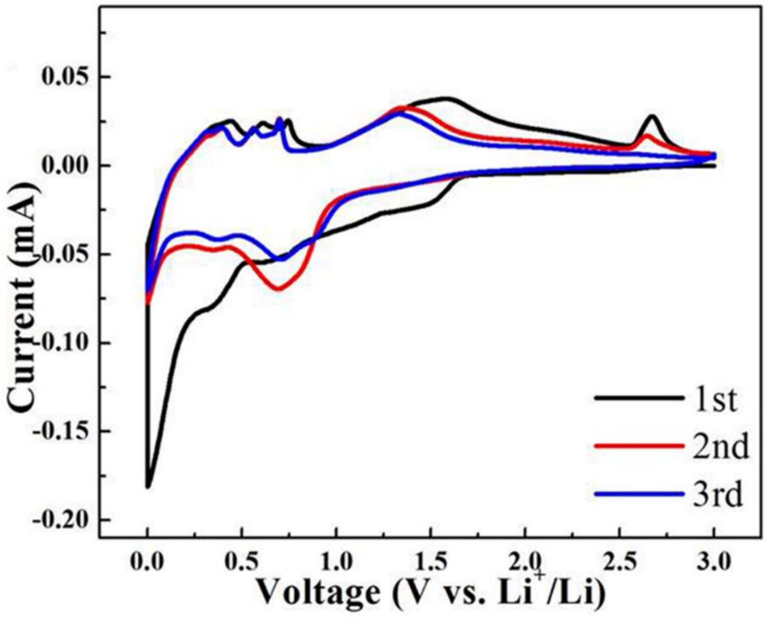

Figure S9. CV curves of pristine ZnO films electrodes for the initial three cycles at the scan rate of 0.3 mV/s in the voltage range of 0-3 V.

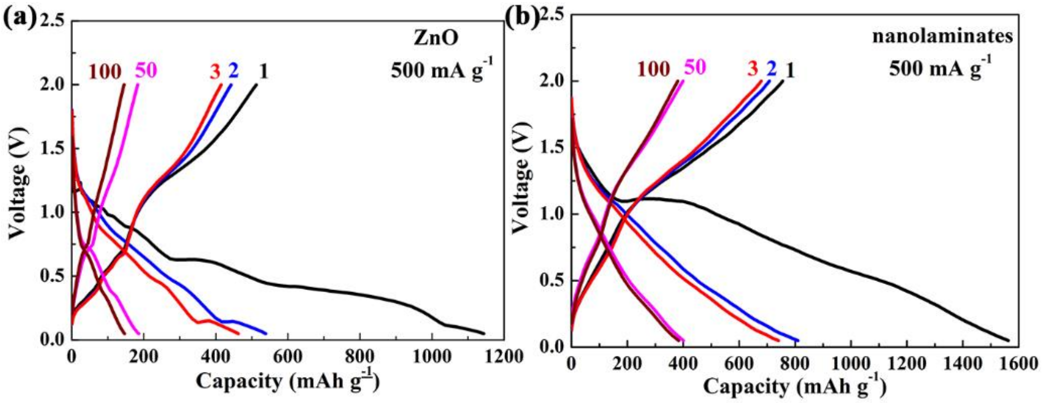

Figure S10. Discharge/charge profiles of (a) ZnO and (b) ZnO/TiO_2 nanolaminates at 500 mA g^{-1} within 0.05-2.0 V.

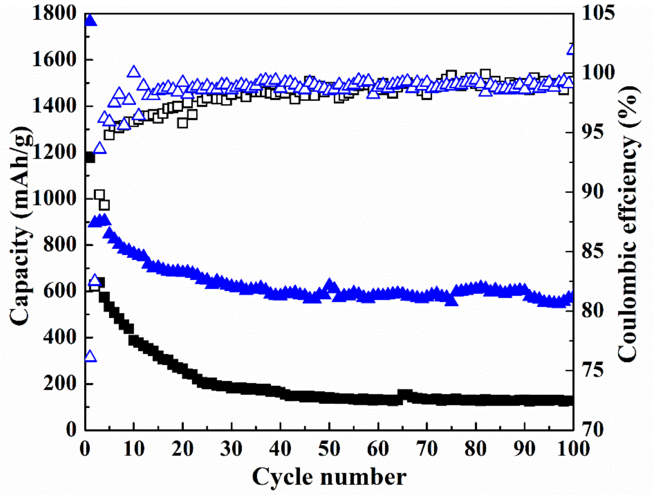

Figure S11. Cycling performance and CE of ZnO/TiO_2 nanolaminates and pristine ZnO films at 200 mA/g for 100 cycles in the potential range of 0.05-2.0 V.

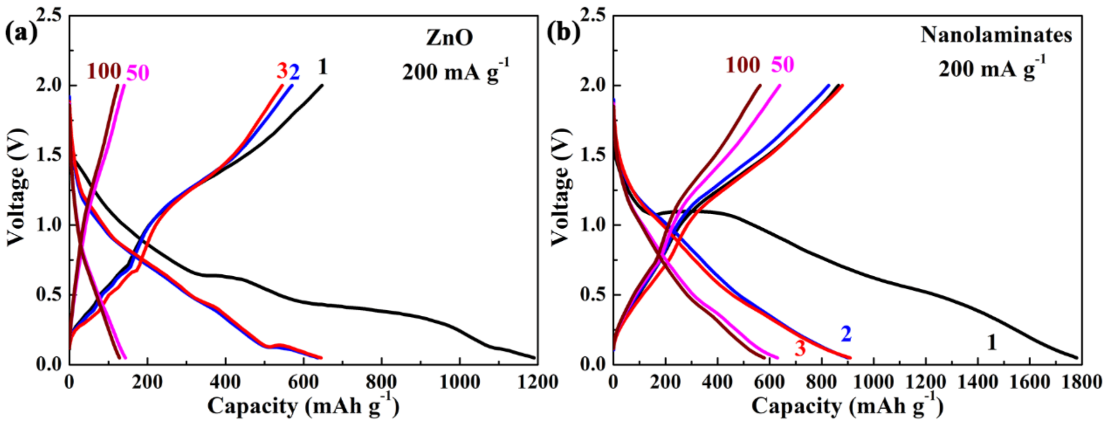

Figure S12. Discharge/charge profiles of (a) ZnO and (b) ZnO/TiO_2 nanolaminates at 200 mA g^{-1} within 0.05-2.0 V.

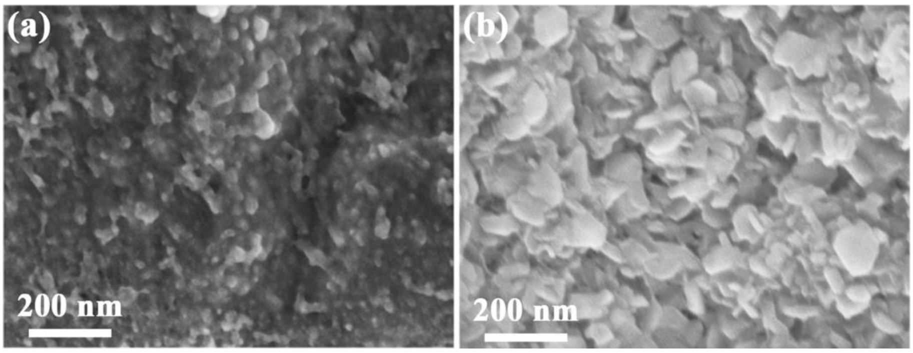

Figure S13. SEM images of (a) pristine ZnO films and (b) ZnO/TiO_2 nanolaminates after 100 cycles charging-discharging at 500 mA g^{-1}.

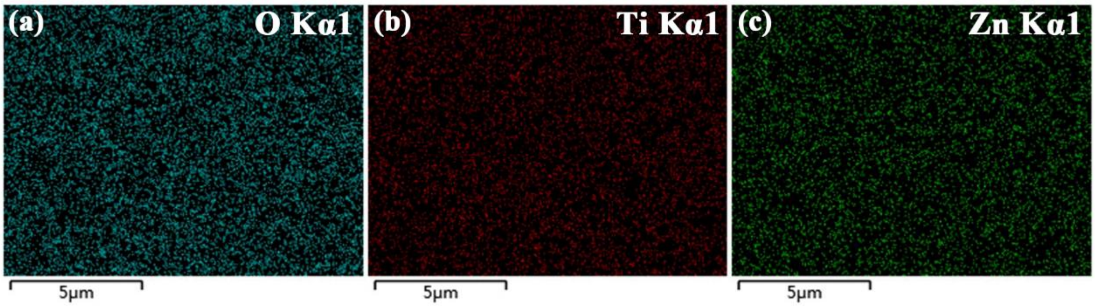

Figure S14. EDS mapping results of ZnO/TiO_2 nanolaminates after 100 cycling of 500 mA g^{-1}.

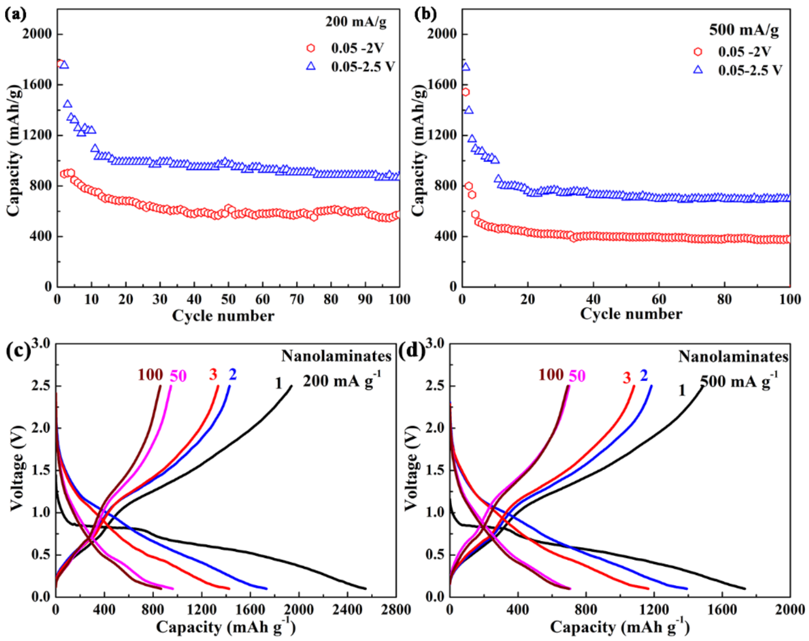

Figure S15. Cycling performance of ZnO/TiO_2 nanolaminates in the potential range of 0.05-2.0 V and 0.05-2.5 V. (a) 200 mA g^{-1} and (b) 500 mA g^{-1}. Discharge/charge profiles of ZnO/TiO_2 nanolaminates at within 0.05-2.5 V with 200 mA g^{-1} and (b) 500 mA g^{-1}.

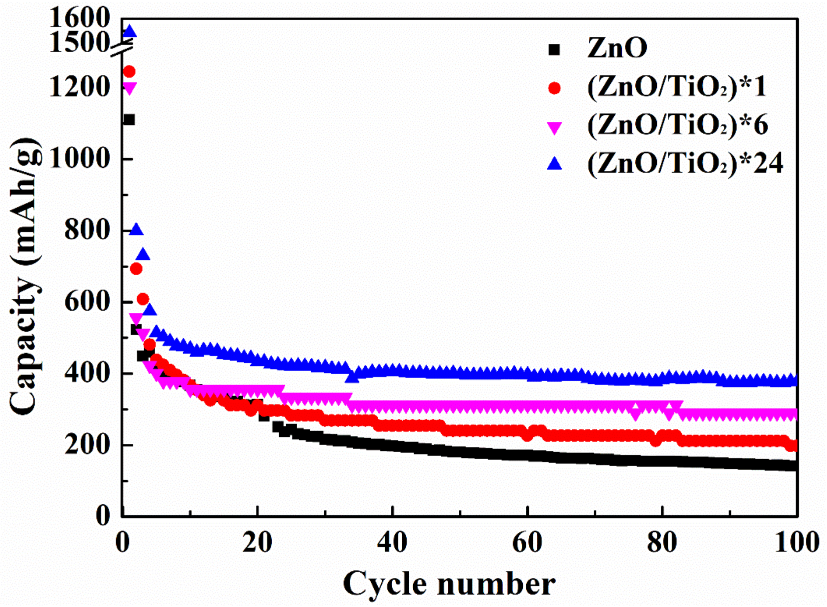

Figure S16. Cycling performance of different ZnO/TiO_2 nanolaminates based anode.

We also explored the performance of (ZnO/TiO_2)*1 and (ZnO/TiO_2)*6 nanolaminate by fixing the total deposition cycles of 624 cycles ZnO and 624 cycles TiO_2. Please see Fig. S14, it can be seen that the nanolaminate structure with more layers (thinner ZnO thickness) possesses the better cycling stability.
